# The Influence of Transmission-Based and Moral-Based HIV Stigma Beliefs on Intentions to Discriminate Among Ward Staff in South Indian Health Care Settings

**DOI:** 10.1007/s10461-022-03755-w

**Published:** 2022-07-01

**Authors:** Wayne T. Steward, Krishnamachari Srinivasan, Tony Raj, Elsa Heylen, Laura Nyblade, Amanda Mazur, Dhinagaran Devadass, Matilda Pereira, Maria L. Ekstrand

**Affiliations:** 1 Center for AIDS Prevention Studies, Department of Medicine, University of California San Francisco, San Francisco, CA USA; 2 St. Johns Research Institute, Bangalore, India; 3 International Development Group, Global Health Division, RTI International, DC Washington, USA; 4 Center for AIDS Prevention Studies, Division of Prevention Science, University of California, San Francisco, 550 16th Street, 3rd Floor, San Francisco, CA 94143 USA

**Keywords:** HIV, Stigma, Intervention

## Abstract

HIV stigma is comprised of several beliefs, including transmission fears and moral judgments against affected communities. We examined the relationships among HIV-related stigma beliefs, endorsement of coercive measures for people living with HIV (PLWH), and intentions to discriminate. We sought to understand to what degree the different stigma beliefs shape support for restrictive policies and discriminatory intentions. Data were drawn from the baseline assessment of DriSti, a cluster randomized controlled trial of an HIV stigma reduction intervention in Indian healthcare settings (NCT02101697). Participants completed measures assessing transmission fears and moral judgments of HIV, endorsement of coercive measures against PLWH (public disclosure of HIV status, refusal of healthcare services, marriage and family restrictions, required testing, and sharing of HIV information in a clinic), and intentions to discriminate against PLWH in professional and personal settings. We utilized multivariate regression modeling with backward elimination to identify the coercive measures and behavioral intentions most strongly associated with moral judgments. 1540 ward staff members completed the assessment. Participants had relatively high perceptions of transmission fears (*M* = 1.92, *SD* = 0.79) and moral judgments (*M* = 1.69, *SD* = 0.83); endorsed more intentions to discriminate in professional (*M* = 6.54, *SD* = 2.28) than personal settings (*M* = 2.07, *SD* = 1.49), and endorsed approximately half of all coercive measures (*M* = 9.47, *SD* = 2.68). After controlling for transmission fears, perceptions of stronger moral judgments against PLWH were significantly associated with higher endorsement of coercive measures related to refusing services (*β* = 0.10, *t* = 4.14, *p* < 0.001) and sharing patients’ HIV status in clinics (*β* = 0.07, *t* = 3.04, *p* = 0.002), as well as with stronger behavioral intentions to discriminate in professional settings (*β* = 0.05, *t* = 2.20, *p* = 0.022). HIV stigma interventions for hospital-based ward staff in India need to focus on both transmission fears and moral judgments that underlie prejudicial beliefs. While the moral judgments are not technically related to risk in a hospital setting, our findings suggest that personnel will continue to discriminate in their professional work so long as these beliefs bear on their decisions and actions.

**Supplementary Information:**

The online version contains supplementary material available at 10.1007/s10461-022-03755-w.

## Background

Stigma against human immunodeficiency virus (HIV) leads people to delay care, which compromises their health and increases the potential for further transmission of the virus [1, 2]. It also shapes the attitudes of providers who deliver services [3, 4], and leads to denials of care or mistreatment in medical settings [5]. Like other parts of the world [6–8], these deleterious effects have been observed in India, which is estimated to have 2,348,000 people living with HIV [9] and where stigma is recognized as a barrier to achieving HIV treatment goals [10]. In a large survey of physicians, nurses, and ward staff in Mumbai and Bengaluru, we found strong endorsement of coercive policies and intentions to discriminate against PLWH. The impact of stigma was especially high among nurses and ward staff, over 75% of whom agreed that women with HIV should not be allowed to have children and that men with HIV should not be allowed to marry. Over 70% of ward staff stated that they would discriminate against people with HIV in a hospital setting, even in situations where the risk of encountering bodily fluids was low [11]. Male gender, younger age, unmarried relationship status, lower education, and not personally knowing anyone with HIV were all associated with greater endorsement of coercive policies and/or intentions to discriminate among the ward staff. In a parallel survey conducted with PLWH living in the local communities, we found that experiences of discrimination and shame over being HIV-positive were associated with self-reported delays in seeking care [12].

Addressing HIV stigma requires consideration of two distinct underlying beliefs: (1) transmission fears and (2) moral judgments. These dimensions were originally identified in HIV stigma work in western settings [13, 14] and incorporated into stigma research and measurement development in India [15, 16]. Transmission fears principally reflect concerns that HIV can be passed between people easily or casually, which motivates avoidance of PLWH. Moral judgments reflect prejudicial attitudes against groups and communities at high risk of HIV. Such judgments are rooted in opprobrium toward same-gender sexual activity, injection drug use, sex work, and promiscuity. HIV stigma amplifies these existing prejudices and leads to people holding PLWH responsible for their infection.

Although the different beliefs are related to one another and can be measured together [15], they are not identical. Transmission fears are a more rational concern for healthcare workers, given the potential for occupational exposure as a result of bodily fluid exchange. Such beliefs are also more directly addressed through intervention strategies that seek to correct misinformation or lack of knowledge about the disease and available treatments. Prior research suggests, however, that the moral judgments about HIV are equally pronounced, if not even stronger, among those in the healthcare field [17]. Intervening against these judgments is somewhat more complicated, as the beliefs are not rooted in misinformation, but rather driven by personal and cultural disapproval of specific groups. A recent systematic review of HIV stigma reduction interventions in healthcare settings found that a majority focused on provision of information, while fewer than half included components such as contact with PLWH, which can raise empathy and potentially counteract moral judgments that lead to blame [7]. Furthermore, even when interventions included more diverse components, challenges remained. In one study with Indian nursing students, nearly half of all participants who expressed blame toward PLWH at study outset still did so following intervention [18]. In a study from Bangladesh, upwards of one quarter of all participants continued to believe after intervention that men who have sex with men (MSM) and sex workers engage in immoral behavior, while over 45% continued to state that people get HIV from engaging in irresponsible behavior [19].

These findings raise questions about what potentially is left unaddressed when an intervention does not (or is not able to fully) address the moral dimensions of stigma in healthcare settings. To understand the role of transmission and moral judgments in healthcare settings, we used baseline assessments from a stigma reduction intervention to examine the relationships among HIV-related stigma beliefs, endorsement of coercive measures for PLWH, and intentions to discriminate among ward staff working in hospital settings in India. We hypothesized that moral judgments against HIV would be uniquely associated with both endorsements of coercive measures and intentions to discriminate, over and above any influence of HIV transmission fears.

## Methods

Data for the current cross-sectional analysis were drawn from the baseline assessment of the DriSti Study, a cluster randomized control trial of an HIV stigma reduction intervention for nursing students and hospital ward staff in India (trial registration number NCT02101697) [20–23]. The intervention involved individually completed tablet-based sessions and an in-person group session with a PLWH. Because the data for this analysis were drawn exclusively from the baseline assessments conducted with ward staff prior to intervention activities and do not report the trial results, the intervention and the nursing student participants are not discussed further [21, 23]. The study received approval from the Institutional Ethical Review Board of St. John’s Medical College and Hospital and the Institutional Review Board of the University of California, San Francisco. The research was also cleared by the Indian Council of Medical Research. All participants gave written informed consent in accordance with the Declaration of Helsinki.

### Participant Recruitment

Ward staff were recruited at 38 hospitals in Bengaluru, Delhi, Mangalore, and Mysore, India, between September 2014 and March 2018. To be eligible, an individual had to have worked at the hospital for at least 1 year, be 18 years of age or older, and be able and willing to consent. Participants completed baseline assessment questionnaires shortly after enrollment. We intentionally chose recruitment sites in the public/governmental, private non-profit, and private for-profit sectors. Eligible ward staff were individuals hired to serve as assistants on the hospital floors. They typically perform manual tasks, such as transporting patients and samples, running errands (e.g., replenishing bedside supplies), attending to patients’ personal hygiene, and assisting with activities such as ambulating, turning, or positioning patients.

We directed recruitment efforts at all ward staff at the facilities, drawing a sample from eligible individuals who elected to participate. We initially contacted the superintendent of each facility for permission to recruit at the site. A project team member then attended ward staff meetings and read a description of the study. Interested individuals were invited to approach the project team member. All participants completed study activities at a convenient time that did not conflict with work obligations. We recruited at different times of the day to capture those working shifts in the morning, afternoon, and early evenings. We were not able to conduct study activities in the late-night hours, although ward staff working at those times were permitted to take part in the study at another time of the day. All participants provided written informed consent. Those who completed a survey during work hours were not compensated to avoid conflict with hospital policies prohibiting additional payment while on duty; otherwise, they received reimbursement for travel expenses.

### Baseline Survey

The survey was interviewer-administered and completed on a tablet in Kannada, Hindi, or English. Computer-assisted personal interviewing technology displayed assessment questions and allowed participant responses to be entered. The survey was programmed with logic checks and question routing instructions to minimize errors [22]. The technology also allowed the survey to be tailored for ward staff. All interviewers were required to complete a training prior to interactions with participants. Training included reviewing survey items to ensure the interviewers understood the questions being asked and then practicing administration with mock participants. Subsequently, newly trained interviewers began administering surveys under supervision from a masters-level project manager and a PhD-level co-investigator. Interviewers had to be certified as demonstrating sufficient competency with the survey tool before being allowed to collect data on their own.

Participant responses were stored on the tablets and subsequently imported into an electronic database. The survey measured several constructs of interest.

#### Perceived Stigma

We used a validated 10-item measure that we developed to assess the perceived prevalence of HIV stigmatizing beliefs and behaviors among ward staff [15]. It was adapted from prior research in western settings on the roles of transmission fears and moral judgments in manifestations of HIV stigma [13, 14]. Five items captured HIV transmission fears (e.g., “Among ward staff, how many would not share dishes or glasses with a person with HIV”); the other five captured moral judgments (e.g., “Among ward staff, how many think people with HIV are paying for their karma or sins?”). Participants used a four-item response scale (0 = no one; 3 = most ward staff). An overall scale score was derived by averaging responses to all 10 items (Cronbach’s ⍺ = 0.85). Subscale scores were derived by averaging responses to the five items that captured transmission fears (⍺ = 0.82) and five that captured moral judgments (⍺ = 0.84). It should be noted that, while this measure technically assessed beliefs about stigmatizing attitudes among other ward staff, we have found previously that such perceptions were related to a person’s own beliefs and behaviors (e.g., personal endorsement of stigma beliefs) [15]. This is not necessarily surprising, as perceptions of what peers are thinking ultimately reflect what a respondent perceives to be normative, hence driving decisions and actions.

#### Endorsement of Coercive Measures

Participants completed a 21-item measure that assessed agreement with coercive strategies to control the behavior of PLWH. A portion of the items was taken from a measure used in a prior study [11]. We added additional items to capture other coercive beliefs identified in prior work and during formative activities to develop the intervention. A principal components analysis with varimax rotation indicated that the items loaded onto six factors: four captured disclosure (e.g., “People should have the right to decide whether or not to disclose their HIV status to their providers”); four captured the right to deny services (e.g., “Health care workers should be able to refuse to treat a person with HIV”); four captured beliefs about punishment being deserved (e.g., “People who get HIV through drugs have gotten what they deserved”); four focused on marriage and family (e.g., “Women with HIV infection should not be allowed to marry”); three captured beliefs about mandatory testing (e.g., “All female sex workers should be required to be tested for HIV”); and two captured beliefs about forcibly sharing HIV information in healthcare settings (e.g., “A patient who has HIV should have a clearly visible label on their medical files identifying them as HIV positive”). Participants responded to all items using a four-point response scale (1 = strongly disagree; 4 = strongly agree).

The four items that assessed beliefs about punishment were conceptually similar to the moral judgment items of the felt stigma scale and were not used for these analyses. A total measure score for the remaining 17 items was calculated by assigning one point for each item to which a participant responded “agree” or “strongly agree” (endorsing a coercive measure) and then summing across items (⍺ = 0.62). Several items were originally written so that agreement with them reflected non-stigmatizing beliefs. Responses to these items were reverse coded prior to scoring. Similar procedures were used to create scores for the subsets of items associated with each of the five factors (disclosure: ⍺ = 0.68; deny services: ⍺ = 0.57; marriage and family: ⍺ = 0.40; mandatory testing: ⍺ = 0.55; share HIV information in healthcare settings: ⍺ = 0.54).

#### Intentions to Discriminate

We assessed both personal and professional intentions to discriminate, using scales we developed in prior research [11]. For the seven items capturing personal intentions, participants were asked to use a four-point response scale (1 = strongly disagree; 4 = strongly agree) to indicate the degree to which they endorsed statements that described discriminatory behaviors outside of work (e.g., “I would refuse to live in a house next to one occupied by person with HIV”). Three of the items were written to describe non-discriminatory behaviors (e.g., would eat from same plate as a person living with HIV) and were reverse coded prior to analysis. A total measure score was calculated by assigning one point for each item to which a participant responded “agree” or “strongly agree,” indicating endorsement of a stigmatizing belief, and then summing across items (⍺ = 0.53).

For professional intentions to discriminate, participants responded to eight items that described activities that ward staff members regularly complete (e.g., cleaning up a patient’s bodily fluids, dressing a wound). We developed the measure by initially taking items used in our prior research [11]. We then met with hospital personnel to characterize the kinds of activities in which ward staff engage. We divided the tasks into higher or lower risk and created a set of additional items drawn from each risk stratum. For each item of the measure, a participant was asked to describe what he or she would do if assigned a task with a PLWH. Answer options were: refuse or try to get someone else to do the task, complete the task but do so in a way that avoids touching the patient, complete the task but use additional precautions, and perform the task as one would do with any other patient. The first three answer options reflected intentions to discriminate, as professional guidelines for Indian hospital settings recommend the use of universal precautions with all patients, regardless of health status. A total measure score was calculated by assigning one point for each item to which a participant endorsed a discriminatory choice of action and then summing across items (⍺ = 0.89).

#### Demographics

Items captured participants’ age, gender, religion, marital status, monthly income, and highest level of education. These demographic factors were selected because they are potentially associated with stigma and its impacts [11].

### Analyses

Descriptive statistics were generated for all variables. These included frequencies and proportions for categorical variables, along with means and standard deviations for continuous variables. We initially explored bivariate associations among stigma, coercive measures, and intentions to discriminate using Pearson product-moment correlations. We also tested for differences by demographic characteristics. To identify the specific coercive measures and behavioral intentions most strongly associated with the stigma subscales of moral judgments and transmission fears, we constructed two multivariate ordinary least squares (OLS) regression models. In each model, one set of stigma beliefs (either transmission fears or moral judgments) was regressed onto coercive measures and intentions to discriminate, while the other set of stigma beliefs, along with any demographic characteristics that were associated with stigma in bivariate analyses, were entered as covariates (i.e., in one model, transmission fears served as a predictor of moral judgments, while in the other model, moral judgments served as a predictor of transmission fears). We then used backward elimination to remove non-significant predictors until each of the final models consisted only of significant predictors.

## Results

A total of 1540 ward staff members completed the baseline assessment. As reflected in Table 1, nearly two-thirds (64%) were female, 83% were Hindu, and 70% were married. The average age was 40 (*SD* = 9.79). Participant income ranged from less than 5000 rupees/month (4.7%) to more than 20,000 rupees/month (28%). For purposes of analyses, we divided participants approximately evenly based on whether they made more than 15,000 rupees/month. Participants’ education also varied from less than 5 years (indicating only partial primary schooling; 13%) to having some college education (2%). We divided the responses into three groups reflective of little (less than 7 years of schooling), moderate (7–10 years), or higher levels of education (more than 10 years), with a majority (85%) having 10 or fewer years. Table 2 displays the means and standard deviations for the perceived stigma, coercive measures, and behavioral intentions overall and divided by whether participants made more than 15,000 rupees/month or not, which emerged as the demographic characteristic with the strongest relationship to the variables of interest. Overall, participants had relatively high perceptions of transmission fears (*M* = 1.92, *SD* = 0.79 on a subscale running 0–3) and moral judgments (*M* = 1.69, *SD* = 0.83 on a subscale running 0–3), endorsed more intentions to discriminate in professional (*M* = 6.54, *SD* = 2.28 on a scale running 0–8) than personal settings (*M* = 2.07, *SD* = 1.49 on a scale running 0–7), and endorsed approximately half of all coercive measures (*M* = 9.47, *SD* = 2.68 for the overall scale running 0–17). More specifically, participants endorsed all or nearly all of the items related to mandatory HIV testing (*M* = 2.89, *SD* = 0.39, on a subscale running 0–3), while endorsing few of the items related to refusing services (*M* = 0.79, *SD* = 1.00 on a subscale running 0–3). Relative to participants making 15,000 rupees/month or less, individuals making more than 15,000 rupees/month had significantly higher perceptions of transmission fears (*F* = 8.22, p = 0.004) and significantly lower perceptions of moral judgments (*F* = 7.70, p = 0.006); were significantly more likely to endorse coercive policies related to mandatory disclosure of HIV status (*F* = 136.34, p < 0.001) and mandatory HIV testing (*F* = 9.28, *p* = 0.002) but significantly less likely to endorse policies to refuse services to PLWH (*F* = 30.51, *p* < 0.001); and were significantly more likely to indicate intentions to discriminate in professional settings (*F* = 6.90, *p* = 0.009).

**Table 1:** Demographic characteristics of the participants

	Number	Percent
Gender		
Male	561	36.4
Female	979	63.6
Religion		
Hindu	1281	83.2
Christian	206	13.4
Other	53	3.4
Marital status		
Single	198	12.6
Married	1083	70.3
Formerly married	259	16.8
Income		
≤ 15,000 rupees/month	857	55.6
> 15,000 rupees/month	683	44.3
Education		
≤ 7 years	610	39.6
7–10 years	706	45.8
> 10 years	224	14.5

	Mean	St. Dev
Age (range: 18–62)	39.48	9.79

**Table 2:** Descriptive statistics of participants’ perceived stigma, endorsement of coercive measures, and intentions to discriminate (n = 1540)

Variable	All		≤ 15,000 Rupees/month		> 15,000 Rupees/month	
	Mean	Std Dev.	Mean	Std Dev.	Mean	Std Dev.
Felt stigma (full scale)	1.81	0.69	1.81	0.68	1.81	0.69
Moral judgments	1.69	0.83	1.75	0.82	1.63	0.83
Transmission fears	1.92	0.79	1.86	0.76	1.98	0.81
Coercive measure (full scale)	9.47	2.68	9.13	2.65	9.89	2.66
Disclosure	1.93	0.79	1.51	1.51	2.46	1.68
Refuse service	0.79	1.00	0.92	1.08	0.64	0.86
Marriage and family	2.44	1.14	2.42	1.12	2.47	1.18
Required testing	2.89	0.39	2.87	0.43	2.93	0.32
HIV status shared in clinic	1.41	0.73	1.42	0.72	1.40	0.73
Behavioral intentions—personal	2.07	1.49	2.13	1.50	2.00	1.46
Behavioral intentions—professional	6.54	2.28	6.40	2.39	6.70	2.12

Table 3 presents the correlations among the stigma, coercive measures, and behavioral intention measures. Perceived moral judgments and transmission fears were significantly correlated with one another (*r* = 0.44, *p* < 0.010). Overall, perceived stigma, encompassing both moral judgments and transmission fears, was significantly and positively associated with greater endorsements of coercive measures (*r* = 0.14, *p* < 0.010) and intentions to discriminate in the personal (*r* = 0.15, *p* < 0.010) and professional domains (*r* = 0.15, *p* < 0.010). However, coercive measures focused on disclosure showed a significant negative association with stigma (*r* = − 0.08, *p* < 0.010). As noted earlier, we separately found that perceptions of moral judgments and transmission fears were associated with income (see Table 2). In additional we found a gender effect for moral judgments, with women endorsing such perceptions at significantly higher levels than men (Men: *M* = 1.63, *SD* = 0.79; Women: *M* = 1.73, *SD* = 0.85; *F* = 6.22, *p* < 0.010). Perceived moral judgments and transmission fears did not associate reliably with participants’ religion, marital status, education, or age.

**Table 3:** Bivariate correlations among perceived stigma, endorsement of coercive measures, and intentions to discriminate (n = 1540)

Study measure	PS-all	PS-moral	PS-transmit	CM-all	CM-Dis	CM-RS	CM-MF	CM-RT	CM-SHIV	BI-Pers	BI-Prof
Perceived stigma (PS)—all items	1.00										
PS—moral judgments	0.86**	1.00									
PS—transmission fears	0.84**	0.44**	1.00								
Coercive measures (CM)—all	0.14**	0.11**	0.13**	1.00							
CM—disclosure (Dis)	− 0.08**	− 0.08**	− 0.06*	0.68**	1.00						
CM—refuse service (RS)	0.16**	0.17**	0.11**	0.54**	0.90**	1.00					
CM—marriage and family (MF)	0.22**	0.15**	0.23**	0.53**	0.02	0.14**	1.00				
CM—required testing (RT)	0.06*	0.05	0.06*	0.24**	0.05	0.01	0.08**	1.00			
CM—share HIV info in clinic (SHIV)	0.12**	0.13**	0.08**	0.44**	0.08**	0.19**	0.10**	0.10**	1.00		
Behavioral intentions (BI)—personal	0.15**	0.14**	0.12**	0.28**	0.07*	0.40**	0.12**	0.03	0.15**	1.00	
BI—professional	0.15**	0.12**	0.14**	0.20**	0.07*	0.09**	0.18**	0.15**	0.10**	0.11**	1.00

*p < 0.05; **p < 0.01

Table 4 displays the results of our multivariate models. In the top half of the table, we present the model predicting moral judgments. The findings reveal that, after accounting for the effects of transmission fears (*β* = 0.42, *t* = 18.37, *p* < 0.001) and income (*β* = − 0.07, *t* = − 3.09, *p* < 0.001), stronger perceptions of moral judgments against PLWH were significantly associated with higher endorsement of coercive measures related to refusing services (*β* = 0.10, *t* = 4.14, *p* < 0.001) and sharing HIV status in clinics (*β* = 0.07, *t* = 3.04, *p* = 0.002), as well as with stronger behavioral intentions to discriminate in professional settings (*β* = 0.05, *t* = 2.20, *p* = 0.022). Gender, coercive measures related to marriage and family, and intentions to discriminate in personal settings were not retained in the model. In the lower half of Table 4, we display the model predicting transmission fears. After controlling for the effects of moral judgments (*β* = 0.41, *t* = 17.98, *p* < 0.001) and income (*β* = 0.12, *t* = 4.96, *p* < 0.001), stronger perceptions of transmission fears were significantly associated with greater endorsement of coercive measures related to restrictive policies for marriage and family (*β* = 0.15, *t* = 6.61, *p* < 0.001), as well as with higher behavioral intentions to discriminate in personal (*β* = 0.05, *t* = 2.17, *p* = 0.031) and professional (*β* = 0.05, *t* = 2.27, *p* = 0.023) settings. Coercive measures related to refusal of services and sharing of HIV status in clinics were not retained in this model. As a point of contrast evidenced in both models, coercive measures related to disclosure of HIV status were significantly associated with *lower* perceived moral judgments (*β* = − 0.05, *t* = − 2.27, *p* = 0.023) and transmission fears (*β* = − 0.07, *t* = − 2.81, *p* = 0.005).

**Table 4:** Multivariate model of constructs associated with perceived HIV stigma beliefs

	B	Std Err	Beta	t	p value
Moral judgments					
Covariates					
Transmission fears	0.445	0.024	0.423	18.37	< 0.001
Income	− 0.123	0.040	− 0.074	− 3.087	0.002
Coercive measure					
Disclosure	− 0.027	0.012	− 0.054	− 2.271	0.023
Right to refuse service	0.080	0.019	0.097	4.137	< 0.001
Share HIV info in clinic	0.079	0.026	0.070	3.041	0.002
Behavioral intentions					
Professional	0.019	0.008	0.053	2.300	0.022
Transmission fears					
Covariates					
Moral judgments	0.390	0.022	0.411	17.98	< 0.001
Income	0.183	0.037	0.116	4.958	< 0.001
Coercive measure					
Disclosure	− 0.031	0.011	− 0.066	− 2.810	0.005
Marriage and family	0.104	0.016	0.151	6.613	< 0.001
Behavioral intentions					
Personal	0.026	0.012	0.049	2.165	0.031
Professional	0.018	0.008	0.052	2.273	0.023

## Discussion

Our findings help to elucidate the associations among stigma beliefs, support for coercive measures, and intentions to discriminate. They suggest that stronger perceptions of HIV stigma are associated with discriminatory intentions and support of coercive measures. This aligns with the substantial body of evidence of the deleterious impact of stigma on the lives of PLWH [10–12, 24, 25]. Our findings additionally show how the two types of stigma beliefs specifically relate to support for stigmatizing measures and behavior. Perceptions of transmission fears and moral judgments were associated with intentions to discriminate in professional settings and with reduced endorsement of coercive measures to require disclosure of HIV status. Transmission fears showed degrees of unique association with coercive measures to restrict marriage and family life and with intentions to discriminate in personal settings. By contrast, moral judgments showed degrees of unique association with coercive measures pertaining to sharing information in medical settings and refusing services to PLWH.

These findings demonstrate how beliefs that underlie HIV-related stigma influence the kinds of policy choices and personal actions that people believe are appropriate. Our participants almost universally indicated that HIV testing should be mandated, which accounts for its failure to emerge as a unique correlate of either moral judgments or transmission fears in the multivariate modeling. Transmission fears led to stronger willingness to sacrifice the marital and family life of PLWH to prevent spread of the virus. Participants who scored higher on the measure of transmission fears also endorsed greater intentions to discriminate in both the personal and professional domains, consistent with (inaccurate) beliefs that HIV is easily transmissible. These findings represent a key challenge for HIV stigma reduction interventions in healthcare settings. The pattern of beliefs points to a simplified understanding of infection control, one that insists that the person living with HIV bear pronounced and pervasive sacrifices to bring the risk of transmission to near zero because the healthcare worker believes PLWH pose a substantive risk to hospital staff. This is not a reasonable or productive balancing of responsibilities, as overly restrictive testing and family life policies, coupled with overt discrimination against PLWH, may drive those most at risk away from testing and treatment [25]. Such findings have been shown, for example, in research on pregnant women living with HIV, who reported that fears of stigmatization acted as a barrier to services to prevent mother-to-child transmission [26]. A stigma reduction intervention thus must encourage a participant to engage with a more sophisticated risk–benefit analysis, recognizing that larger public health objectives related to near zero transmission are better achieved when policies and practices enable some degree of risk among PLWH (e.g., coupling with others who are infected) but which is offset by enhanced engagement with the healthcare system—and ultimately reduced infectivity—among those living with HIV.

The findings also point to the unique role that perceived moral judgments may play in the kinds of policies and practices favored by hospital ward staff and others who work in healthcare settings. We found that perceptions of such judgments led to greater endorsement of policies that would allow PLWH to be denied services and to have their HIV status forcibly shared in healthcare settings. Given observed challenges faced by HIV interventions in fully addressing stigma rooted in moral judgments [18, 19], these findings point to the kinds of behaviors that might still be evidenced by providers and clinical staff even after exposure to stigma reduction interventions. Such behaviors pose substantive concerns for the care of PLWH. Prior research has shown, for example, that concerns about forced disclosure can decrease engagement in care [27]. Additionally, feelings of blame, if ultimately endorsed by PLWH themselves, contribute to psychological distress [15, 28]. The challenge is figuring out how to fully address moral judgments, which are tied to “beliefs in a just world” [29] (those who get a disease did something to deserve that disease) and opprobrium against certain groups of people or activities (sex work, infidelity, drug use, homosexuality). These are trickier areas for intervention, as they do not necessarily relate to misinformation, but rather to deeply held personal convictions and religious tenets.

Interestingly, an unexpected finding in our results may provide insights into how perceptions of moral judgments could be successfully tackled. We found there to be a negative association between perceived stigma and endorsement of coercive public disclosure policies. We cannot rule out entirely the possibility of measurement error but given that participants’ responses to other questions were consistent with expected relationships among constructs, we think it unlikely. A reasonable explanation for the result is that those who hold stigmatizing beliefs may be particularly concerned about the potential impact that a forced disclosure policy would have on their own wellbeing. In India and elsewhere, family members, friends, and caregivers of those who are stigmatized can, themselves, become the target of “courtesy stigma” (stigma by association) [30]. They become judged and mistreated merely for their close association with a disliked group. To the degree that participants in our study see courtesy stigma as a reasonable possibility, there would be concerns about how public disclosure policies might circle back to the participants themselves (e.g., if a family member were to test positive for HIV). This provides an angle on which stigma interventions can build. The risk of courtesy stigma creates a common vulnerability with patients. Such affiliation is known to motivate greater emphatic responses, which in turn can reduce stigmatizing behaviors [31].

Our findings are tempered by several limitations. First, the analyses presented here are correlational in nature. We thus cannot draw formal conclusions about causality. Second, the data are limited to hospital ward staff. We do not have reasons to believe that the fundamental patterns observed in our findings would differ for other healthcare providers and staff, but further research will be needed to verify this assumption. Third, we are not able to account for certain characteristics of ward staff that might influence stigma beliefs. In particular, the total years of experience as a hospital ward staff and a complete history of interactions with PLWH both in and outside of work could influence transmission fears or moral judgments. Fourth, data for this study were collected between 2014 and 2018. We do not have reasons to believe that the distinctions between transmission fears and moral judgments, nor the implications of them, would have changed between then and now. However, it is possible that the relative prevalence of each may have shifted.

## Conclusions

HIV stigma interventions for hospital-based ward staff in India need to focus on both the transmission fears and moral judgments that underlie prejudicial beliefs. While the moral judgments are not technically related to risk in a hospital setting, our findings suggest that personnel will continue to discriminate in their professional work so long as these beliefs are allowed to come to bear on their decisions and actions.

## Supplementary Information

Below is the link to the electronic supplementary material.

Supplementary file1 (DOCX 232 KB)

## Data Availability

Data from this study cannot be posted to a public repository as doing so would be inconsistent with the approvals granted by the Indian government. However, de-identified data can be made available to individual investigators upon request to the senior author (MLE).

## Abbreviations

HIV: Human immunodeficiency virus
MSM: Men who have sex with men
OLS: Ordinary least squares
PLWH: People living with HIV
