# Supplementary material for: The Influence of Transmission-Based and Moral-Based HIV Stigma Beliefs on Intentions to Discriminate Among Ward Staff in South Indian Health Care Settings
Source: AIDS Behav. 2022 Jul 1;27(1):189–97. doi: 10.1007/s10461-022-03755-w (PMC9805471; doi:10.1007/s10461-022-03755-w)
Supplement: Supplementary file 1 — Supplementary file1 (DOCX 232 KB) [file 10461_2022_3755_MOESM1_ESM.docx]

##### Measures Used in Manuscript

**Demographics and job-related characteristics**

**D1.**INTERVIEWER: Please note the language in which the interview is primarily being conducted.

| **Language** | **D1. Interview Language** |
| --- | --- |
| Kannada | *1* |
| Hindi | *2* |
| English | *3* |

| **D2.** | **What is your age?** [INTERVIEWER: A number is required. If the participant doesn’t know, please try to get approximate answer. | ** (years*)*    -9 Declined to Answer |
| --- | --- | --- |
| **D3.** | **What is the participant’s sex?** [INTERVIEWER: it is not necessary to ask this question to participants. Check appropriate box without asking the question. | 0*.* Male  1. Female  2.Transgender  -9. Decline to answer |

| **D4. What is your religion?** [INTERVIEWER: Do not read the responses. Have the participant tell you his or her religion, then check the appropriate box.] | | | ***Check Appropriate Box*** |
| --- | --- | --- | --- |
|  |  | Hindu | *1* |
|  |  | Muslim | *2* |
|  |  | Sikh | *3* |
|  |  | Buddhist | *4* |
|  |  | Christian | *5* |
|  |  | Jain | *6* |
|  |  | Jewish | *7* |
|  |  | Paris/ Zoroastrian | *8* |
|  |  | None | *9* |
|  |  | Declined to answer | *-9* |

| **D5.** | **What is your current marital status?** [INTERVIEWER: Do not read the responses.  Have the participant tell you marital status, then check the appropriate box.] | | | | | |
| --- | --- | --- | --- | --- | --- | --- |
|  |  |  |  |  | ***Check Appropriate Box*** |  |
|  |  |  |  | Currently Married | *1* |  |
|  |  |  |  | Never Married/Single | *2* |  |
|  |  |  |  | Divorced | *3* |  |
|  |  |  |  | Separated | *4* |  |
|  |  |  |  | Widow/Widower | *5* |  |
|  |  |  |  | Deserted | *6* |  |
|  |  |  |  | Declined to answer | *-9* |  |

| **D6.** | **What is your estimate for the combined monthly income of your household? Please include all different sources of income for all those whom you live with. Would you say it is? [***INTERVIEWER: read response categories.]* | | | |  |
| --- | --- | --- | --- | --- | --- |
| 5,000 or less | 1 |  |  |  |  |
| 5,001 – 10,000 | 2 |  |  |  |  |
| 10,001 – 15,000 | 3 |  |  |  |  |
| 15,001 – 20,000 | 4 |  |  |  |  |
| More than 20,000 | 5 |  |  |  |  |
| *Declined to answer* | *-9* |  |  |  |  |

| **D7. WARD STAFF ONLY: What is the highest level of education you completed?** *[INTERVIEWER:* Do not read the response options. Mark the appropriate box, based on participant’s response.] |  |
| --- | --- |

| *≤ 4 years* | *1* |
| --- | --- |
| *5-7 years* | *2* |
| *8-10 years* | *3* |
| *Vocational training* | *4* |
| *Second PUC/Junior College* | *5* |
| *Some degree college* | *6* |
| *Other* | *7* |
| *Declined to answer* | *-9* |

**Behavioral Intentions (personal)**

**I am going to read you some more statements that different people have made. Please tell me how much you agree or disagree with each statement as it pertains to your personal life, apart from your job.**

[Interviewer: Circle the option selected by the participant; Do not read the “Don’t Know” and “Declined to answer” options to the participant. Use (Visual No. 2) to explain the response options and rate the appropriate response].

|  |  | **Strongly disagree** | **Disagree Somewhat** | **Agree Somewhat** | **Strongly Agree** | *Don’t know* | *Declined to answer* |
| --- | --- | --- | --- | --- | --- | --- | --- |
| **BIP1.** | **I would not seek services from a doctor or nurse living with HIV** | 1 | 2 | 3 | 4 | *-8* | *-9* |
| **BIP2.** | **I would refuse to live in a house next to one occupied by a person with HIV** | 1 | 2 | 3 | 4 | *-8* | *-9* |
| **BIP3.** | **I would eat food from the same plate that had been previously used by a person with HIV** | 1 | 2 | 3 | 4 | *-8* | *-9* |
| **BIP4.** | **I would feel comfortable about feeding by hand a person with HIV** | 1 | 2 | 3 | 4 | *-8* | *-9* |
| **BIP5.** | **I would take care of children with HIV or children whose parents are HIV-positive** | 1 | 2 | 3 | 4 | *-8* | *-9* |
| **BIP6.** | **I would avoid visiting a relative with HIV** | 1 | 2 | 3 | 4 | *-8* | *-9* |
| **BIP7.** | **I feel that people with HIV should be treated in separate clinics/hospitals so that they can avoid infecting others** | 1 | 2 | 3 | 4 | *-8* | *-9* |

**Behavioral Intentions (professional)**

**Now I’m going to read to you some tasks that [nurses/ward staff] may have to perform at work, and ask you how you feel about these task and how you do them with different types of patients. If you do not actually do the task that I am reading in your job, please tell me how you *would* feel and what you *would* do if you were told to do it.**

[INTERVIEWER: be sure and read each response option to the participant. Circle the option selected by the participant. Use Visual No 3 to explain the ‘worried’ response options and rate the appropriate response. Go through all columns before moving to next row, so do a, b, c, d for each task.

**Suppose in the course of your work you had to do the following tasks.**

| **WARD STAFF** | **(a) If this was a patient with HIV, how worried would you feel about [name task]?**  (use visual 3) | **(b) What do you think you would do when you were told to [name task] on this patient with HIV?** | **(c) Now if this was a patient with TB instead of HIV, then how worried would you feel about [name task]?**  (use visual 3) | **(d) What do you think you would do when you were told to [name task] on this TB patient?** |
| --- | --- | --- | --- | --- |
| **BIJ11.** **Cleaning up a patient’s bodily fluids like blood and contaminated linen** | - 1. Very worried   2. Somewhat worried   3. A little worried   4. Not at all worried   5. Declined to answer | (a) I would refuse or try to get someone else to do it.  (b) I would do it, but in such a way that I would avoid touching the patient as much as possible.  (c) I would do it, but with extra precautions (such as double gloves).  (d) I would do it as I would with any other patient.  (e) Declined to answer | - 1. Very worried   2. Somewhat worried   3. A little worried   4. Not at all worried   5. Declined to answer | - 1. I would refuse or try to get someone else to do it.   2. I would do it, but in such a way that I would avoid touching the patient as much as possible.   3. I would do it, but with extra precautions (such as double gloves).   4. I would do it as I would with any other patient.   5. Declined to answer |
| **BIJ13. Dress a patient’ s wound** | (a) Very worried  (b) Somewhat worried  (c) A little worried  (d) Not at all worried  (e) Declined to answer | (a) I would refuse or try to get someone else to do it.  (b) I would do it, but in such a way that I would avoid touching the patient as much as possible.  (c) I would do it, but with extra precautions (such as double gloves).  (d) I would do it as I would with any other patient.  (e) Declined to answer | (a) Very worried  (b) Somewhat worried  (c) A little worried  (d) Not at all worried  (e) Declined to answer | (a) I would refuse or try to get someone else to do it.  (b) I would do it, but in such a way that I would avoid touching the patient as much as possible.  (c) I would do it, but with extra precautions (such as double gloves).  (d) I would do it as I would with any other patient.  (e) Declined to answer |
| **BIJ14. Assist in the operating theater, including with labor and delivery, on a patient.** | (a) Very worried  (b) Somewhat worried  (c) A little worried  (d) Not at all worried  (e) Declined to answer | (a) I would refuse or try to get someone else to do it.  (b) I would do it, but in such a way that I would avoid touching the patient as much as possible.  (c) I would do it, but with extra precautions (such as double gloves).  (d) I would do it as I would with any other patient.  (e) Declined to answer | (a) Very worried  (b) Somewhat worried  (c) A little worried  (d) Not at all worried  (e) Declined to answer | (a) I would refuse or try to get someone else to do it.  (b) I would do it, but in such a way that I would avoid touching the patient as much as possible.  (c) I would do it, but with extra precautions (such as double gloves).  (d) I would do it as I would with any other patient.  (e) Declined to answer |
| **BIJ15. Assist a patient with his or her personal hygiene needs, such as bathing** | (a) Very worried  (b) Somewhat worried  (c) A little worried  (d) Not at all worried  (e) Declined to answer | (a) I would refuse or try to get someone else to do it.  (b) I would do it, but in such a way that I would avoid touching the patient as much as possible.  (c) I would do it, but with extra precautions (such as double gloves).  (d) I would do it as I would with any other patient.  (e) Declined to answer | (a) Very worried  (b) Somewhat worried  (c) A little worried  (d) Not at all worried  (e) Declined to answer | (a) I would refuse or try to get someone else to do it.  (b) I would do it, but in such a way that I would avoid touching the patient as much as possible.  (c) I would do it, but with extra precautions (such as double gloves).  (d) I would do it as I would with any other patient.  (e) Declined to answer |
| **BIJ16. Transport a patient** | (a) Very worried  (b) Somewhat worried  (c) A little worried  (d) Not at all worried  (e) Declined to answer | (a) I would refuse or try to get someone else to do it.  (b) I would do it, but in such a way that I would avoid touching the patient as much as possible.  (c) I would do it, but with extra precautions (such as double gloves).  (d) I would do it as I would with any other patient.  (e) Declined to answer | (a) Very worried  (b) Somewhat worried  (c) A little worried  (d) Not at all worried  (e) Declined to answer | (a) I would refuse or try to get someone else to do it.  (b) I would do it, but in such a way that I would avoid touching the patient as much as possible.  (c) I would do it, but with extra precautions (such as double gloves).  (d) I would do it as I would with any other patient.  (e) Declined to answer |
| **BIJ17. Take a patient’s blood pressure** | (a) Very worried  (b) Somewhat worried  (c) A little worried  (d) Not at all worried  (e) Declined to answer | (a) I would refuse or try to get someone else to do it.  (b) I would do it, but in such a way that I would avoid touching the patient as much as possible.  (c) I would do it, but with extra precautions (such as double gloves).  (d) I would do it as I would with any other patient.  (e) Declined to answer | (a) Very worried  (b) Somewhat worried  (c) A little worried  (d) Not at all worried  (e) Declined to answer | (a) I would refuse or try to get someone else to do it.  (b) I would do it, but in such a way that I would avoid touching the patient as much as possible.  (c) I would do it, but with extra precautions (such as double gloves).  (d) I would do it as I would with any other patient.  (e) Declined to answer |
| **BIJ19. Transporting a patient’s lab specimens or samples** | (a) Very worried  (b) Somewhat worried  (c) A little worried  (d) Not at all worried  (e) Declined to answer | (a) I would refuse or try to get someone else to do it.  (b) I would do it, but in such a way that I would avoid touching the patient as much as possible.  (c) I would do it, but with extra precautions (such as double gloves).  (d) I would do it as I would with any other patient.  (e) Declined to answer | (a) Very worried  (b) Somewhat worried  (c) A little worried  (d) Not at all worried  (e) Declined to answer | (a) I would refuse or try to get someone else to do it.  (b) I would do it, but in such a way that I would avoid touching the patient as much as possible.  (c) I would do it, but with extra precautions (such as double gloves).  (d) I would do it as I would with any other patient.  (e) Declined to answer |
| **BIJ20. Taking care of the dead body of a patient** | (a) Very worried  (b) Somewhat worried  (c) A little worried  (d) Not at all worried  (e) Declined to answer | (a) I would refuse or try to get someone else to do it.  (b) I would do it, but in such a way that I would avoid touching the patient as much as possible.  (c) I would do it, but with extra precautions (such as double gloves).  (d) I would do it as I would with any other patient.  (e) Declined to answer | (a) Very worried  (b) Somewhat worried  (c) A little worried  (d) Not at all worried  (e) Declined to answer | (a) I would refuse or try to get someone else to do it.  (b) I would do it, but in such a way that I would avoid touching the patient as much as possible.  (c) I would do it, but with extra precautions (such as double gloves).  (d) I would do it as I would with any other patient.  (e) Declined to answer |

**Perceived Stigma**

**Now I am going to read to you some statements that other ward staff have made about HIV and people with HIV. These are only the opinions of some people and they may or may not reflect your own. There are no correct answers, but we are interested in your thoughts. After I read to you each statement, please give me your best guess regarding how many nurses/ward staff share that opinion.**

**For example, if I were to read the following…**

**How many other ward staff enjoy drinking tea? Would you say “No one”, “Very few ward staff”, “Some ward staff”, or “Most ward staff”?**

[INTERVIEWER: read the questions and response options to the participant. Do not read the “Don’t Know” and “Declined to answer” options to the participant. Use (Visual No. 4) to explain the response options and mark the appropriate response*]*

|  |  | **No One** | **Very few**  **ward attendants** | **Some**  **ward attendants** | **Most**  **ward attendants** | *Don’t know* | *Decline to answer* |  |
| --- | --- | --- | --- | --- | --- | --- | --- | --- |
| **FS1** | | **Among ward attendants, how many would not want a person with HIV to hold their young child?** | 0 | 1 | 2 | 3 | *-8* | *-9* |
| **FS2** | | **Among ward attendants, how many would not want a person with HIV to feed their young child?** | 0 | 1 | 2 | 3 | *-8* | *-9* |
| **FS3** | | **Among ward attendants, how many would not share dishes or glasses with a person with HIV?** | 0 | 1 | 2 | 3 | *-8* | *-9* |
| **FS4** | | **Among ward attendants, how many would not want a person with HIV cooking for them?** | 0 | 1 | 2 | 3 | *-8* | *-9* |
| **FS5** | | **Among ward attendants, how many would avoid visiting the homes of people with HIV?** | 0 | 1 | 2 | 3 | *-8* | *-9* |
| **FS6** | | **Among ward attendants, how many think that if someone has HIV then he/she has done wrong behaviors?** | 0 | 1 | 2 | 3 | *-8* | *-9* |
| **FS7** | | **Among ward attendants, how many think people with HIV are paying for their karma or sins?** | 0 | 1 | 2 | 3 | *-8* | *-9* |
| **FS8** | | **Among ward attendants, how many think that people with HIV deserve their disease?** | 0 | 1 | 2 | 3 | *-8* | *-9* |
| **FS9** | | **Among ward attendants, how many think that people with HIV should feel guilty about it?** | 0 | 1 | 2 | 3 | *-8* | *-9* |
| **FS10** | | **Among ward attendants, how many think that people with HIV have brought shame upon their families?** | 0 | 1 | 2 | 3 | *-8* | *-9* |

**Endorsement of coercive measures for PLHIV**

**I am going to read you some more statements that different people have made. Please tell me how much you agree or disagree with each statement.**

[Interviewer: Circle the option selected by the participant; Do not read the “Don’t Know” and “Declined to answer” options to the participant. Use (Visual No. 2) to explain the response options

|  |  | **Strongly disagree** | **Disagree Some**  **what** | **Agree Some**  **what** | **Strongly Agree** | *Don’t know* | *Decline to answer* |
| --- | --- | --- | --- | --- | --- | --- | --- |
| **CM1** | **Health care workers should be able to refuse to treat a person with HIV.** | 1 | 2 | 3 | 4 | *-8* | *-9* |
| **CM2** | **People with HIV should have the right to decide whether or not to disclose their HIV status to their health care providers** | 1 | 2 | 3 | 4 | *-8* | *-9* |
| **CM3** | **People who get HIV through drugs have gotten what they deserve** | 1 | 2 | 3 | 4 | *-8* | *-9* |
| **CM4** | **Employers should be able to fire a worker living with HIV and is well enough to work** | 1 | 2 | 3 | 4 | *-8* | *-9* |
| **CM5** | **Women with the HIV infection should *NOT* be allowed to marry.** | 1 | 2 | 3 | 4 | *-8* | *-9* |
| **CM6** | **A landlord should have the right to refuse to rent a room to a person with HIV** | 1 | 2 | 3 | 4 | *-8* | *-9* |
| **CM7** | **People who get HIV through a blood transfusion have gotten what they deserve** | 1 | 2 | 3 | 4 | *-8* | *-9* |
| **CM8** | **A man with HIV should have the right to decide whether or not to disclose his HIV status to his wife** | 1 | 2 | 3 | 4 | *-8* | *-9* |
| **CM9** | **A women with HIV should have the right to decide whether or not to disclose her HIV status to her husband.** | 1 | 2 | 3 | 4 | *-8* | *-9* |
| **CM10** | **All female sex workers should be required to be tested for HIV.** | 1 | 2 | 3 | 4 | *-8* | *-9* |
| **CM11** | **People who get HIV through sex have gotten what they deserve.** | 1 | 2 | 3 | 4 | *-8* | *-9* |
| **CM12** | **Women with HIV should be allowed to have children.** | 1 | 2 | 3 | 4 | *-8* | *-9* |
| **CM13** | **People with HIV should have the right to decide whether or not to disclose their HIV status to their family (other than their spouse)** | 1 | 2 | 3 | 4 | *-8* | *-9* |
| **CM14** | **People who get HIV from their husbands or wives have gotten what they deserve.** | 1 | 2 | 3 | 4 | *-8* | *-9* |
| **CM15** | **Children with HIV should *not* be allowed to attend school as they may infect others** | 1 | 2 | 3 | 4 | *-8* | *-9* |
| **CM16** | **Men with the HIV infection should NOT be allowed to marry.** | 1 | 2 | 3 | 4 | *-8* | *-9* |
| **CM17** | **All patients undergoing surgery should be required to be tested for HIV.** | 1 | 2 | 3 | 4 | *-8* | *-9* |
| **CM18** | **All medical personnel who perform surgery should be required to be tested for HIV regularly.** | 1 | 2 | 3 | 4 | *-8* | *-9* |
| **CM19** | **A patient who has HIV should have a clearly visible label on their medical files, identifying them as HIV positive.** | 1 | 2 | 3 | 4 | *-8* | *-9* |
| **CM20** | **Other patients in the ward have a right to know if a patient is HIV positive.** | 1 | 2 | 3 | 4 | *-8* | *-9* |
| **CM21** | **People with HIV should only be allowed to marry other people with HIV.** | 1 | 2 | 3 | 4 | *-8* | *-9* |
